# A case of giant nipple adenoma

**DOI:** 10.1186/s40792-024-01869-y

**Published:** 2024-03-25

**Authors:** Shuko Ono, Masumi Tanaka, Yasuteru Yoshinaga, Toshihiko Satou, Mikiko Aoki

**Affiliations:** 1 Department of General Thoracic, Breast, and Pediatric Surgery, Fukuoka University Hospital, 7-45-1 Nanakuma, Jounann-Ku, Fukuoka, 814-0180 Japan; 2 Department of Pathology, Fukuoka University School of Medicine, Fukuoka University Hospital, 7-45-1 Nanakuma, Jounann-Ku, Fukuoka, 814-0180 Japan

**Keywords:** Nipple, Giant nipple, Breast tumor, Erosion, Paget’s disease

## Abstract

**Background:**

Nipple adenoma is a relatively rare benign disease. Clinically, it often presents with nipple erosions, and it should be differentiated from Paget’s disease.

**Case presentation:**

The patient was a 63-year-old woman who complained of a lump in her left nipple for more than 30 years. Computed tomography performed for screening congestive heart failure suggested a left nipple mass of 40 mm in size. Needle biopsy revealed nipple adenoma, and skin biopsy was also performed to confirm the diagnosis. Nipple tumor resection was performed under local anesthesia, and we confirmed that the final diagnosis was nipple adenoma with negative margins. The patient has been free from recurrence for 2 years since the surgery.

**Conclusions:**

We have reported our experience of a case of giant nipple adenoma.

## Background

Nipple adenoma is a relatively rare benign disease that occurs within the nipple or in the milk ducts just below the nipple. Clinically, the disease often presents with nipple erosions, and it should be differentiated from Paget’s disease. Histologically, the lesions consist of small ductal adenomas with a bilayered epithelial and myoepithelial structure, and it should also be differentiated from breast cancer and syringoma. We report a case of giant nipple adenoma.

## Case presentation

**Fig. 1:**
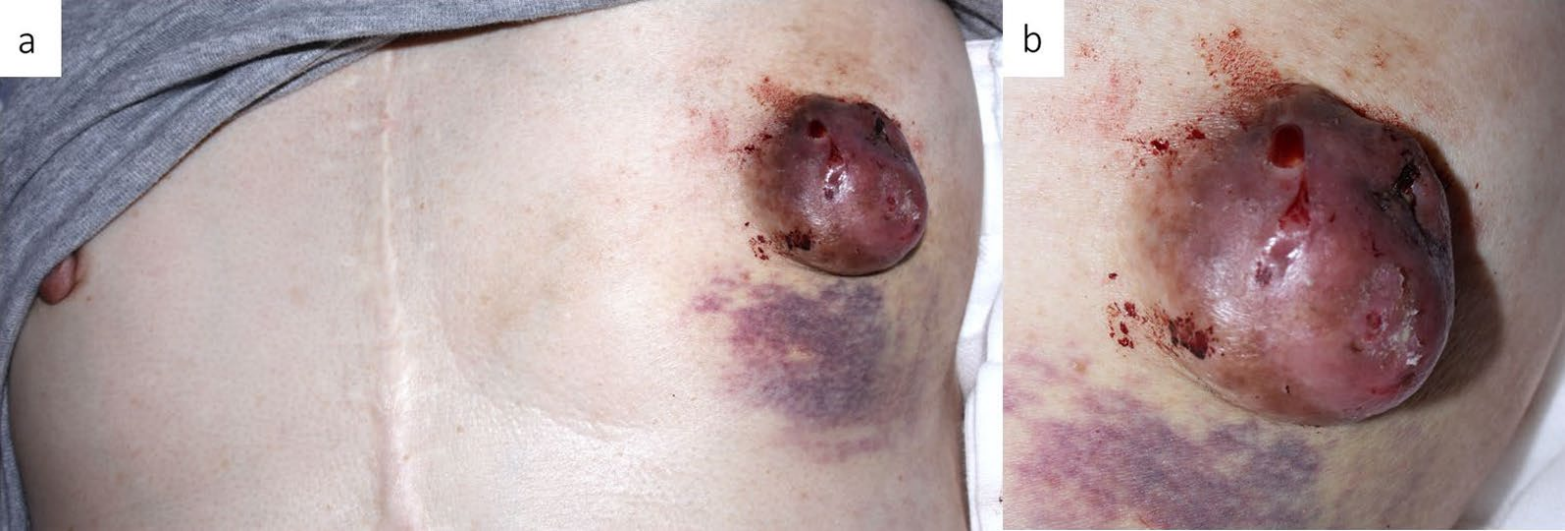
**a** Mass 40 mm in size coinciding with the left nipple. **b** Mass was prone to bleeding

A 63-year-old Japanese woman had been aware of a lump in her left nipple for more than 30 years. Breast cancer was previously ruled out at another hospital, but she did not receive further follow-up. She underwent computed tomography (CT) for screening congestive heart failure and a mass in the left nipple was detected. She was referred to our department on suspicion of breast cancer (Fig. 1).

**Fig. 2:**
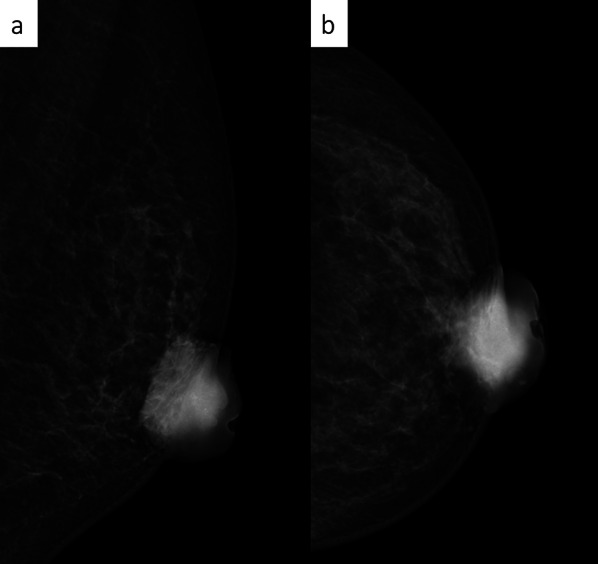
On mammography, a pleomorphic, fine lobulated mass was detected in the left UMLS/OS region. The lesion additionally exhibited conglomerated calcifications, category 4 [**a** Mediolateral Oblique (MLO), **b** Cranio-Caudal (CC)]

**Fig. 3:**
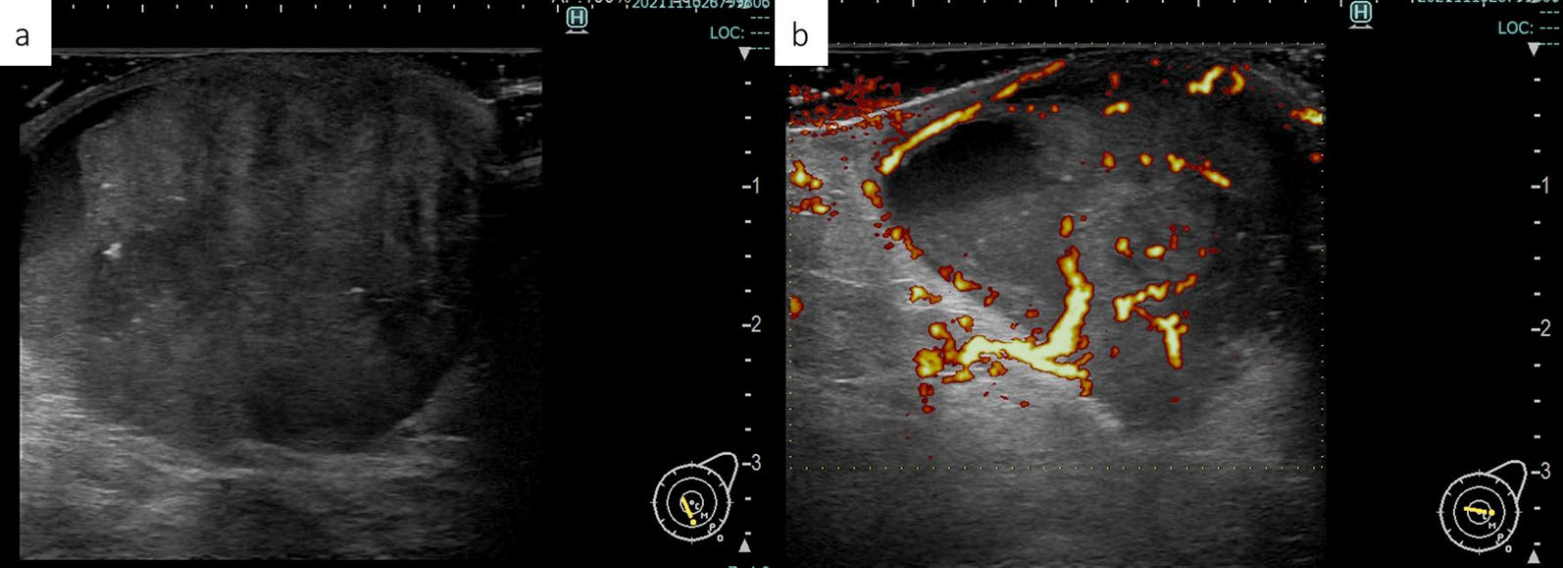
Breast ultrasound uncovered a hypoechoic mass measuring 39 × 31 mm with abundant blood flow in the left nipple area

**Fig. 4:**
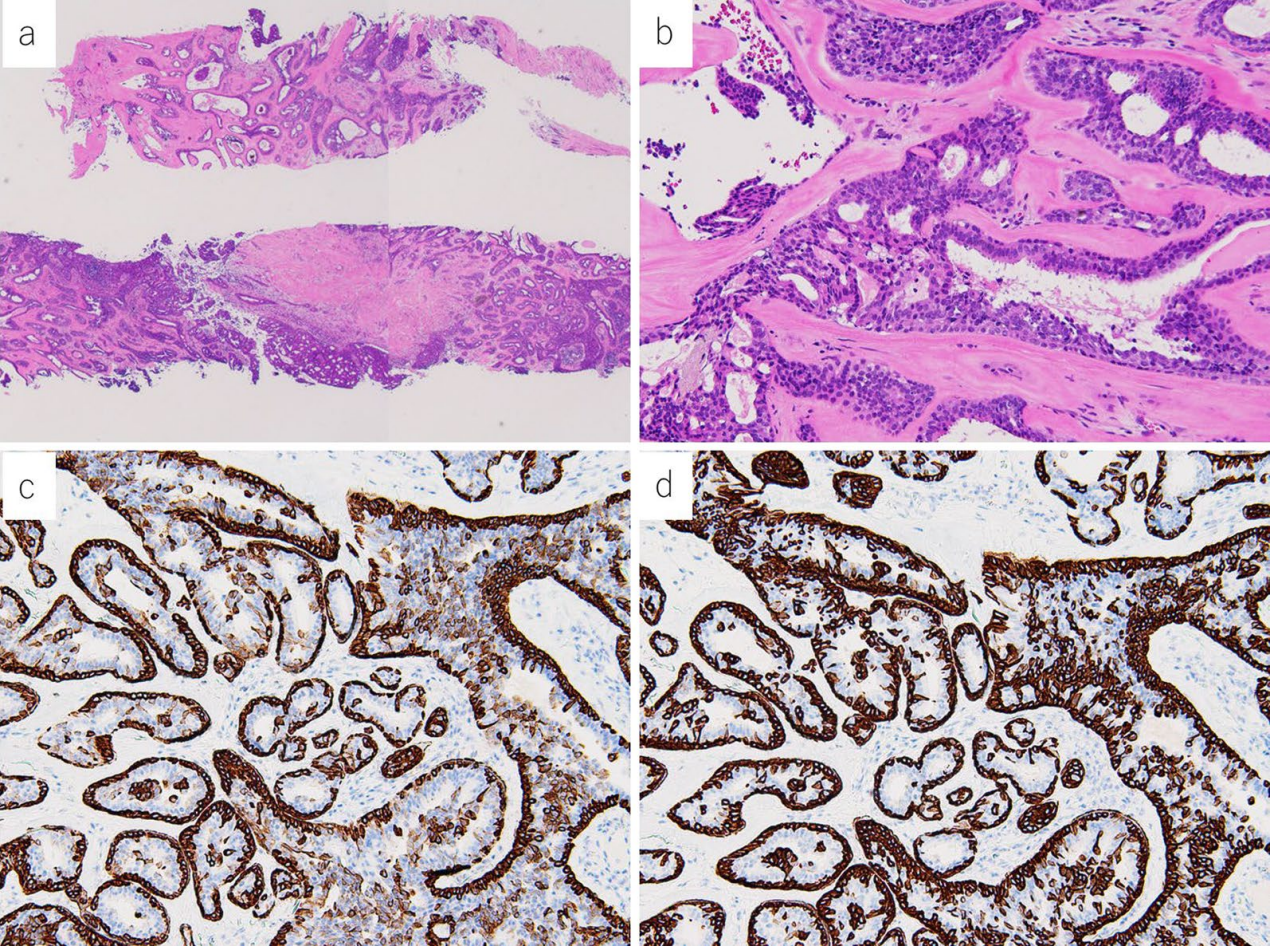
**a** Low-magnification image of hematoxylin and eosin staining. **b** High-magnification image of hematoxylin and eosin staining. **c** CK5/6 was expressed in a mosaic pattern. **d** CK14 was expressed in a mosaic pattern

The left nipple tumor was 40 mm in size without obvious erosion, but the nipple was easily hemorrhagic. CT revealed a heterogeneous internal mass lesion consistent with the nipple area of the left breast. There were no enlarged lymph nodes in the axilla or around the clavicle. Mammography revealed a fine lobulated mass with pleomorphic and conglomerated calcifications consistent in the nipple area, and the lesion was classified as category 4 (Fig. 2). Ultrasonography revealed that the left nipple was occupied by a hypoechoic mass of 39 × 31 mm in size with abundant blood flow (Fig. 3). Echo-guided needle biopsy with a 14G needle was performed and prolifetating atypical. Pathological findings of biopsy specimen showed a proliferating atypical epithelial cells exhibiting adenoductal, cribriform, and cord-like structures were histologically observed. The proliferating epithelium displayed mosaic expression of cytokeratin (CK) 5/6 and CK14. The diagnosis of sclerosing adenopathy and intraductal papilloma was made on the basis of the findings of hyperplasia (Fig. 4). Although needle biopsy was suspicious for sclerosing adenopathy and intraductal papilloma, because of the large size of the tumor, and tendency to bleed easily, breast cancer or Paget’s disease was not completely excluded, thus skin punch biopsy was performed to confirm the diagnosis.

**Fig. 5:**
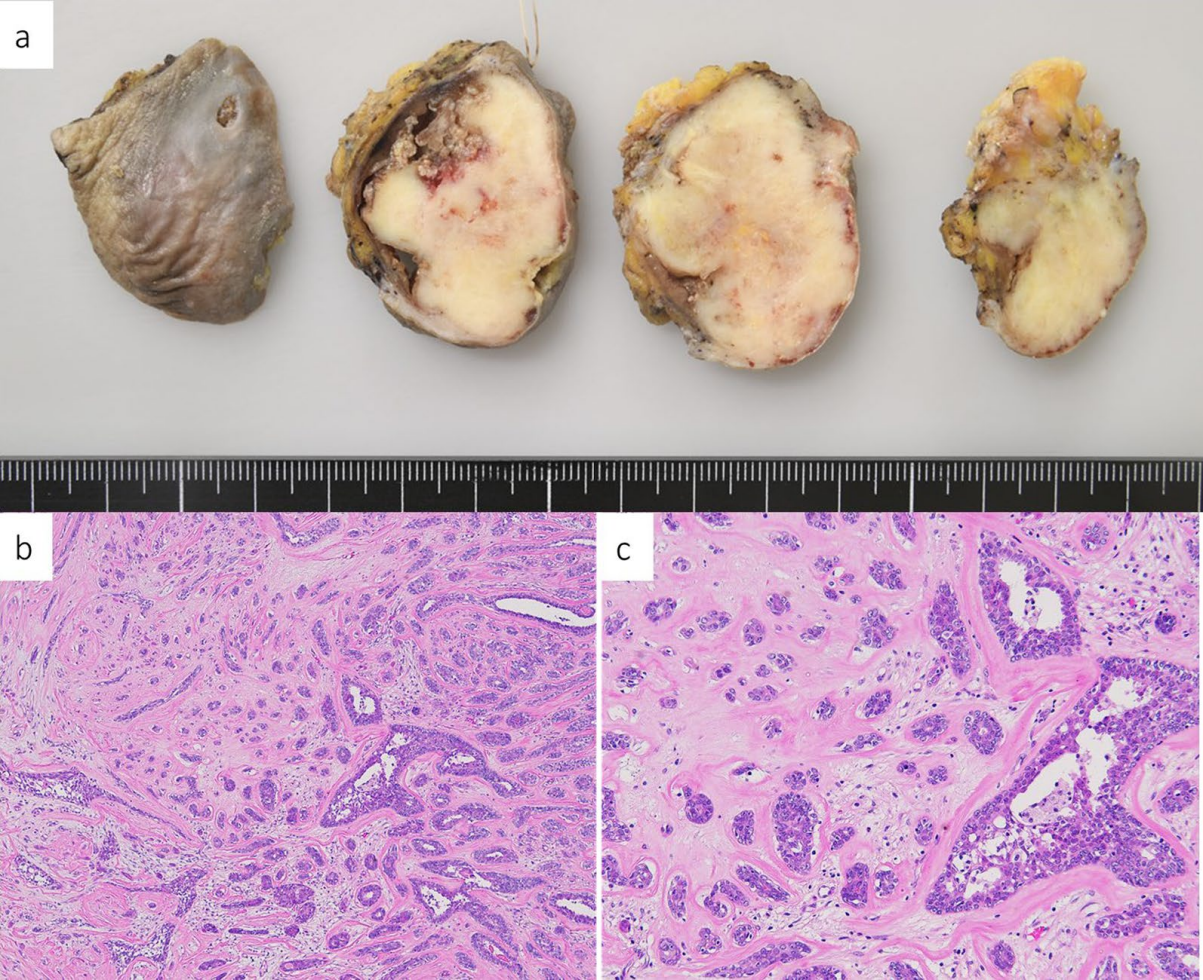
**a** Gross image after formalin fixation. **b** Low-magnification image of hematoxylin and eosin staining. **c** High-magnification image of hematoxylin and eosin staining. The bilayered structure of the epithelium and myoepithelium was preserved with no obvious atypia and Paget’s cells

Proliferation of small glandular ducts was observed in the dermis, and sclerotic stroma was observed in the deep dermis. The bilayered structure of the epithelium and myoepithelium was preserved with no obvious atypia and Paget’s cells. Because the sample was taken from the nipple area, nipple adenoma was strongly suspected. Both needle and skin biopsies revealed no evidence of malignancy, but surgery was performed because of persistent bleeding from the tumor. Considering her general condition, we resected the nipple tumor (40 × 38 × 34 mm) under local anesthesia (Fig. 5).

Post-operative pathology results showed that it maintains continuity with the epidermis and has a varied histology, with findings that may resemble papillomas or sclerosing adenopathy. Keratin cysts and squamous cellularity were also seen. The proliferating epithelium remained bilayered with epithelium and myoepithelium. No malignant findings were identified, and the lesion was diagnosed as nipple adenoma.

The healing of the postoperative wound was uneventful, and the patient is followed up by imaging tests. The patient has remained free from recurrence 2 years since surgery.

## Discussion

Nipple adenomas are benign tumors. These tumors most commonly arise in patients in their 40 s and 50 s, but there are reports of nipple adenomas occurring in children in their 10 s and in elderly patients in their 70 s [1]. In Japan, Sakumoto et al. reported 34 cases in 1996 [2], Sugamata et al. reported 31 cases in 2002 [3], and Uchida et al. reported 60 cases in 2010 [4]. The most common reason for detection was a mass or induration (66%), followed by erosions or ulcers (48%) and nipple discharge (35%) [5–7]. In reports from Western countries, nipple discharge is common, but in Japan, erosions tend to occur more frequently, sometimes making differential diagnosis from Paget’s disease difficult [2, 17]. The size of nipple adenomas reported in Japan ranged from 0.5 to 24 mm (median, 10 mm). Carter et al. reported nipple adenomas ranging in size from 5 to 35 mm [8], but other than the present case, there are no reports of nipple adenomas larger than 35 mm.

In patients with nipple adenoma, the nipple discharge is often bloody, contributing to the suspicion of malignancy. Many cases of nipple erosions are difficult to visualize and evaluate by ultrasonography and mammography, and cases with smooth margins and a uniform internal mass image [9]. It is important to differentiate erosive lesions from Paget’s disease; thus, it is important to deny the presence of Paget’s cells by performing imprint cytology [10] or skin biopsy [11]. Clinical differentiation is difficult because the symptoms are extremely similar to those of Paget’s disease.

Although this tumor exhibits a variety of histological features, it is possible to differentiate it from Paget’s disease by considering the bilayer structure of the epithelium and myoepithelium, the presence of apocrine-forming cells, and other factors, including the localization of the tumor. If necessary, immunohistochemistry can further improve the accuracy of diagnosis [12]. Frequently used myoepithelial markers are p63, h-caldesmon, calponin 1, α-smooth muscle actin, CK5/6 and CD10. The positivity of at least two markers is sufficient for diagnosis [13, 14].

Although 3.6% of papillary adenomas in the nipple are associated with carcinoma, they are often ectopic [15]. Therefore, progression from this disease is not clear, and there are no reports of metastasis or recurrence after resection [16]. In this case, there was no malignant component despite of large tumor size.

The first choice of treatment is lumpectomy combined with biopsy and treatment because of the difficulty in distinguishing benign from malignant lesions histologically [17]. There have been reports of mastectomies performed because of overdiagnosis, and therefore, an adequate preoperative search is necessary to avoid overtreatment [18].

In the present case, a large tumor occupying the left nipple was clinically suspected to be a malignant lesion, but repeated histological examination led to a diagnosis of nipple adenoma. The tumor in this case was completely resected.

## Conclusion

In this case, we experienced a giant nipple adenoma of the breast. Because of its size, we strongly suspected a malignant lesion and performed needle biopsy and skin biopsy to confirm the diagnosis. The tumor occupying the left nipple was completely resected.

## Data Availability

Data sharing is applicable to this article.

## Abbreviations

CT: Computed tomography
CK: Cytokeratin
